# Supplementary material for: Predictive value of red cell distribution width to albumin ratio for acute kidney injury in patients with acute pancreatitis
Source: PLoS One. 2026 Feb 27;21(2):e0341471. doi: 10.1371/journal.pone.0341471 (PMC12948089; doi:10.1371/journal.pone.0341471)
Supplement: S1 Table — (DOCX) [file pone.0341471.s001.docx]

| Characteristics | Odds Ratio (95% CI) | P value |
| --- | --- | --- |
| Gender |  |  |
| Female | 1.0 |  |
| Male | 1.24 (0.84, 1.82) | 0.2774 |
| Age | 1.02 (1.01, 1.03) | 0.0010 |
| Race |  |  |
| Asian | 1.0 |  |
| Black | 1.27 (0.41, 3.95) | 0.6764 |
| White | 2.49 (0.93, 6.63) | 0.0684 |
| Others | 2.10 (0.76, 5.80) | 0.1530 |
| Hospital time (d) | 1.03 (1.02, 1.05) | <0.0001 |
| ICU time (d) | 1.37 (1.24, 1.52) | <0.0001 |
| Sepsis |  |  |
| No | 1.0 |  |
| Yes | 3.56 (2.40, 5.30) | <0.0001 |
| MI |  |  |
| No | 1.0 |  |
| Yes | 1.78 (0.85, 3.71) | 0.1238 |
| CHF |  |  |
| No | 1.0 |  |
| Yes | 4.23 (2.00, 8.94) | 0.0002 |
| COPD |  |  |
| No | 1.0 |  |
| Yes | 1.25 (0.76, 2.06) | 0.3870 |
| Hypertension |  |  |
| No | 1.0 |  |
| Yes | 0.66 (0.45, 0.97) | 0.0340 |
| Diabetes |  |  |
| No | 1.0 |  |
| Yes | 0.93 (0.62, 1.41) | 0.7475 |
| Severe Liver |  |  |
| No | 1.0 |  |
| Yes | 1.75 (0.66, 4.60) | 0.2593 |
| SAPSII | 1.05 (1.04, 1.06) | <0.0001 |
| SOFA | 1.30 (1.22, 1.39) | <0.0001 |
| Invasive Vent |  |  |
| No | 1.0 |  |
| Yes | 4.84 (3.11, 7.53) | <0.0001 |
| CPR |  |  |
| No | 1.0 |  |
| Yes | 1.17 (0.13, 10.59) | 0.8864 |
| HR (tpm) | 1.00 (0.99, 1.01) | 0.3958 |
| Map (mmHg) | 0.99 (0.98, 1.00) | 0.0154 |
| Wbc (10^9/L) | 1.03 (1.00, 1.06) | 0.0339 |
| RDW (10^12/L) | 1.16 (1.03, 1.30) | 0.0120 |
| Plt (10^9/L) | 1.00 (1.00, 1.00) | 0.5949 |
| Cr (mg/dL) | 1.34 (1.09, 1.64) | 0.0046 |
| Alb (g/dL) | 0.64 (0.48, 0.85) | 0.0020 |
| Bun (mmol/dL) | 1.02 (1.01, 1.03) | 0.0022 |
| RAR | 1.35 (1.16, 1.57) | <0.0001 |
| PT (s) | 1.03 (1.00, 1.06) | 0.0918 |
| INR | 1.30 (0.94, 1.80) | 0.1139 |
| Alt (IU/L) | 1.00 (1.00, 1.00) | 0.2952 |
| Ast (IU/L) | 1.00 (1.00, 1.00) | 0.4726 |
| Hb (g/dL) | 1.06 (0.98, 1.14) | 0.1464 |
