# Supplementary figures and images for: Predictive value of red cell distribution width to albumin ratio for acute kidney injury in patients with acute pancreatitis
Source: PLoS One. 2026 Feb 27;21(2):e0341471. doi: 10.1371/journal.pone.0341471 (PMC12948089; doi:10.1371/journal.pone.0341471)

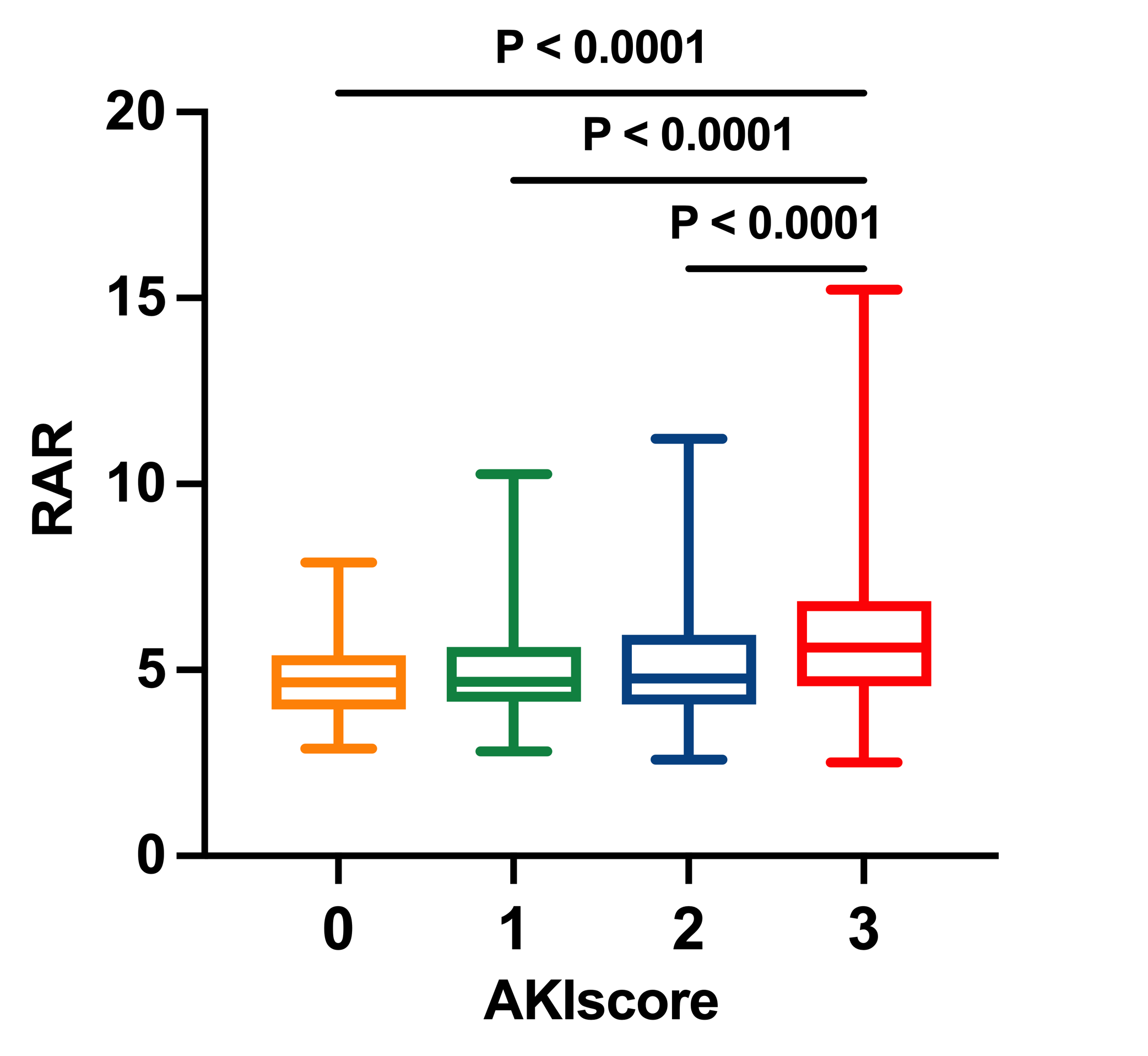

Supplement: S1 Fig — (TIFF) [file pone.0341471.s002.tiff]
