# Supplementary material for: Predictive value of red cell distribution width to albumin ratio for acute kidney injury in patients with acute pancreatitis
Source: PLoS One. 2026 Feb 27;21(2):e0341471. doi: 10.1371/journal.pone.0341471 (PMC12948089; doi:10.1371/journal.pone.0341471)
Supplement: Attachment — Submitted filename: Response to Reviewers.docx [file pone.0341471.s004.docx]

Dear Editors and Reviewers,

Thank you for handling our manuscript (PONE-D-25-29411) and providing constructive comments. We have carefully addressed all reviewers’ comments as follows:

**Reviewer #1**

Q1: It would be appropriate to include a brief introduction with references supporting information on acute pancreatitis and acute renal failure and their adverse outcomes.

**Response:** We sincerely appreciate this insightful suggestion. we have supplemented a brief introduction in the revised manuscript on page 2, lines 34-39, references 1-3: Acute pancreatitis (AP) is a necro-inflammatory disease of the pancreas characterized by abnormal activation of pancreatic enzymes, which triggers systemic inflammation and contributes to poor prognosis^1, 2^. Acute kidney injury (AKI), a common complication in AP patients characterized by a rapid deterioration of renal function, further exacerbates AP-related adverse outcomes and is associated with high mortality^3^.

**References**

1. Lankisch, P. G., Apte, M. & Banks, P. A. Acute pancreatitis. *Lancet* **386**, 85–96 (2015).
2. Peery, A. F. et al. Burden and Cost of Gastrointestinal, Liver, and Pancreatic Diseases in the United States: Update 2018. *Gastroenterology* **156**, 254-272.e11 (2019).
3. Wang, H. E., Muntner, P., Chertow, G. M. & Warnock, D. G. Acute kidney injury and mortality in hospitalized patients. *Am J Nephrol* **35**, 349–355 (2012).

Q2: It would be appropriate to list the strengths and limitations of the study under a separate heading, not within the discussion section.

**Response:** We sincerely thank the reviewer for this valuable suggestion. We agree that presenting the study’s strengths and limitations under a dedicated heading improves clarity and alignment with journal guidelines. In the revised manuscript, we have created a new subsection titled “Strengths and Limitations” after the Discussion (Page 7, line 205).

**Reviewer #2**

Q1. Innovation

The study explores the predictive value of the red cell distribution width to albumin ratio (RAR) for acute kidney injury (AKI) in patients with acute pancreatitis (AP), which represents a notable innovative contribution. To our knowledge, this is the first study to specifically investigate the association between RAR and AKI in critically ill AP patients. The use of RAR as a composite biomarker, integrating the pathophysiological insights of RDW (reflecting oxidative stress and inflammation) and albumin (indicating inflammatory status and nutritional dysregulation), adds novelty compared to single biomarkers (e.g., RDW or albumin alone). Additionally, the establishment of a linear relationship between RAR and AKI risk via restricted cubic splines (RCS) further strengthens the originality of the findings.

**Response:** Thanks for the positive comment.

Q2: Comprehensiveness of Background

The background section provides a solid foundation by contextualizing the clinical significance of AP and AKI, highlighting the role of oxidative stress and inflammation in their pathogenesis. It appropriately reviews the individual roles of RDW and albumin in inflammatory diseases and AP, and logically introduces RAR as a potential composite biomarker. However, the background could be more comprehensive. For instance, a brief discussion of other established or emerging biomarkers for AKI in AP (e.g., neutrophil gelatinase-associated lipocalin, cystatin C) would better position RAR’s unique value. Additionally, while the pathophysiological links between RAR and AKI are outlined, deeper integration of recent studies on the crosstalk between pancreatic inflammation and renal dysfunction could enhance contextual relevance.

**Response:** Thank you for your valuable suggestions. We have revised the background section as follows:

1. We have added a brief discussion of established biomarkers for AP-AKI (e.g., neutrophil gelatinase-associated lipocalin, cystatin C, β2-microglobulin) and compared their limitations with RAR, to better highlight RAR’s unique value. The specific revisions as follows (Page 3, line 67-74): The researches on the prognostic biomarkers of AP-AKI has increased in recent years. Numerous biomarkers have been found to effectively predict AKI development in AP patients, such as neutrophil gelatinase-associated lipocalin (NGAL), β2-microglobulin (β2-MG), and cystatin C^15, 16^. Despite their high sensitivity and specificity, these novel biomarkers are limited by high costs and the need for dynamic monitoring. In contrast, RAR can be rapidly calculated based on routine hematological and biochemical parameters obtained at admission, making it both convenient and affordable.

**References**

15. Wajda, J. et al. Potential Prognostic Markers of Acute Kidney Injury in the Early Phase of Acute Pancreatitis. *Int J Mol Sci* **20**, 3714 (2019).
16. Ruan, Q., Lu, H., Zhu, H., Guo, Y. & Bai, Y. A network-regulative pattern in the pathogenesis of kidney injury following severe acute pancreatitis. *Biomed Pharmacother* **125**, 109978 (2020).

2. We have integrated recent studies on the crosstalk between pancreatic inflammation and renal dysfunction, further elaborating the pathophysiological links between this reciprocal interaction and RAR, to enhance contextual relevance. The specific revisions as follows (Page 2, line 45-53): Notably, recent research highlights the crosstalk between pancreatic inflammation and renal dysfunction in AP. Pancreatic injury releases pro-inflammatory cytokines (e.g., TNF-α, IL-6) and damage-associated molecular patterns (DAMPs) into the systemic circulation. Then these factors trigger renal tubular apoptosis and impair renal microcirculation^6, 7^. Conversely, compromised renal function exacerbates pancreatic injury via accumulated uremic toxins, reduced clearance of inflammatory mediators, and electrolyte disturbances^8^. This interaction emphasizes the need for biomarkers that reflect both local pancreatic inflammation and systemic organ crosstalk.

**References**

6. Zhou, Y. et al. The role of mitochondrial damage-associated molecular patterns in acute pancreatitis. *Biomed Pharmacother* **175**, 116690 (2024).
7. Scurt, F. G., Bose, K., Canbay, A., Mertens, P. R. & Chatzikyrkou, C. [Acute kidney injury following acute pancreatitis (AP-AKI): Definition, Pathophysiology, Diagnosis and Therapy]. *Z Gastroenterol* **58**, 1241–1266 (2020).
8. Shiao, C.-C. et al. Long-term remote organ consequences following acute kidney injury. *Crit Care* **19**, 438 (2015).

Q3: Scientific Rigor of Study Design

The study design is generally sound. It uses a retrospective cohort approach with data extracted from the well-validated MIMIC-IV database, ensuring a relatively large sample size (n=600) and clear inclusion/exclusion criteria. The use of multiple logistic regression (with stepwise adjustment for confounders) and RCS to evaluate the relationship between RAR and AKI is methodologically appropriate. Subgroup analyses further test the stability of the association across key variables (e.g., age, sex, comorbidities).

However, limitations exist:

The retrospective nature introduces inherent selection bias, as unmeasured confounders (e.g., detailed medication history, timing of AP onset) may influence results.

RDW and albumin were only measured at ICU admission, lacking temporal trends, which limits insights into dynamic changes in RAR during disease progression and their impact on AKI.

The reliance on a single database (MIMIC-IV) may restrict generalizability to other populations or healthcare settings.

**Response:** Thank you sincerely for your careful review and insightful comments on our manuscript. We greatly appreciate your recognition of the key limitations of our study, as your summary accurately aligns with the limitations we have already acknowledged in the original text. Your detailed elaboration on unmeasured confounders (e.g., detailed medication history, timing of AP onset) and the specificity of "ICU admission" (rather than general "patient admission") further helps us refine the rigor of our discussion on limitations.

To address your comments and enhance the clarity and accuracy of our manuscript, we have revised the "Limitations" section to explicitly incorporate your valuable points (Page 8, line 215-225): However, this study also has several limitations. Firstly, this was a single center retrospective study with an inherent bias in data collection. Some unmeasured confounding factors, including patients' detailed medication history and the exact onset time of AP, may potentially affect the accuracy of the study results. Second, the data used in this study was derived from a single database, which may limit the generalizability of the study findings. Thirdly, we only measured RDW and serum albumin levels at ICU admission and did not investigate their temporal trends, which could have provided more detailed insights into disease progression. Finally, our findings demonstrate a significant association between RAR and the incidence of AP-AKI. These results should be interpreted as exploratory and necessitate further validation through rigorously designed multicenter prospective studies.

Q4: Reliability of Results

The results are generally reliable. Key findings—including a high AKI incidence (77.3%), a positive association between RAR (as both continuous and categorical variables) and AKI (OR 1.30, 95% CI 1.10-1.53 for continuous; OR 1.75, 95% CI 1.07-2.86 for categorical), and a linear relationship via RCS—are statistically significant and consistent across analyses. ROC curve analysis demonstrating RAR’s superior predictive performance over RDW or albumin alone further supports its utility.

Subgroup analyses show no significant interactions, indicating the robustness of the association. However, the high AKI incidence (77.3%) warrants cautious interpretation, as it may reflect the severe patient population in MIMIC-IV (critically ill AP patients) but should be contextualized with external data to confirm representativeness.

**Response:** We sincerely appreciate your valuable feedback. Your attention to and prudent perspective on the high incidence of acute kidney injury (AKI) (77.3%) have provided crucial insights for us to further refine the interpretation of our study.

Regarding the interpretation of this data, we fully concur with your viewpoint. The participants in this study were all patients diagnosed with acute pancreatitis (AP) from the MIMIC-IV database, and all of them were critically ill patients in the intensive care unit (ICU) setting. Multiple existing studies have demonstrated that critically ill AP patients exhibit a significantly higher incidence of AKI compared to the general AP patient population. This is attributed to the complexity of their condition, intense systemic inflammatory response, and elevated risk of multi-organ involvement—factors that collectively account for the relatively high AKI incidence observed in our study. To a certain extent, this data reflects the disease characteristics of the specific study population within the MIMIC-IV database.

Meanwhile, we are acutely aware of the potential limitations in representativeness inherent in single-database studies. Therefore, in the Discussion section of our manuscript, we have already acknowledged this limitation of the current research. In future studies, we plan to incorporate multi-center data encompassing AP patients with varying degrees of disease severity. This will allow us to further validate the association between the red cell distribution width to albumin ratio (RAR) and AKI incidence, thereby more comprehensively evaluating the applicability and representativeness of RAR across different patient populations.

Q5: Reasonableness of Conclusions

The conclusions are largely consistent with the results. The claim that “high RAR is an independent risk factor for AKI in critically ill AP patients” is supported by multivariate analyses and RCS results. The suggestion that “early assessment of RAR may facilitate risk stratification” is reasonable, given RAR’s accessibility and predictive performance.

Notably, the authors appropriately acknowledge the exploratory nature of the findings and the need for validation in prospective studies, avoiding overinterpretation of causality (which is prudent for a retrospective analysis).

**Response:** Thanks for the positive comment.

Q6: Grammar and Language

The manuscript is generally well-written with clear syntax and consistent use of terminology. Minor issues include:

Occasional awkward phrasing (e.g., “the relationship between RAR and AKI risk in critical ill patients with AP” should be “critically ill”).

Inconsistencies in abbreviation usage (e.g., “CRRT” is defined but occasionally written as “RRT” without clarification in subgroup analyses).

A few typographical errors (e.g., “appliaction” instead of “application” in the Methods section).

**Response:** Thank you very much for your valuable comments and suggestions on our manuscript. We have carefully addressed all the issues raised and made corresponding revisions:

1. For the awkward phrasing, we have corrected "critical ill patients" to "critically ill patients" to ensure the accuracy and fluency of the expression.
2. Regarding the inconsistencies in abbreviation usage, we have standardized the use of "CRRT" throughout the manuscript, especially in the subgroup analyses, and removed the ambiguous use of "RRT" without clarification to maintain consistency.
3. Concerning the typographical error, we have corrected "appliaction" to "application" in the Methods section to eliminate the mistake.

We hope these revisions meet your requirements.

Sincerely,

Chenyang Xu
