# Supplementary material for: Predictive value of red cell distribution width to albumin ratio for acute kidney injury in patients with acute pancreatitis
Source: PLoS One. 2026 Feb 27;21(2):e0341471. doi: 10.1371/journal.pone.0341471 (PMC12948089; doi:10.1371/journal.pone.0341471)
Supplement: Attachment — Submitted filename: Response_to_Reviewers_auresp_2.docx [file pone.0341471.s005.docx]

Dear Editors and Reviewers,

Thank you for handling our manuscript and providing constructive comments. We have carefully addressed the comments as follows:

**Reviewer #1**

Q1: Dear author, I see that you have made the necessary corrections. Thank you. Your article is eligible for publication in its current form.

**Response:** Thank you for reviewing our manuscript and providing valuable comments.

**Reviewer #2**

Q1. Thank you for revising the previous manuscript. However, I believe there is still an issue that needs to be addressed before the article can be published. Specifically, I think several key factors influencing the development of acute kidney injury (AKI) in patients with acute pancreatitis have not been adjusted for in the multivariate analysis. These factors include hematocrit (which is closely associated with the prognosis of patients with acute pancreatitis), fluid resuscitation volume within the first 24 hours, and intra-abdominal pressure—all of which are of great clinical significance. Could you please review the data and consider supplementing the analysis with these factors?

**Response:** Thank you for your comment. We have re-conducted the multivariate analysis as suggested and incorporated hematocrit (Hct) as an additional adjusting variable in Model 3. The results remain consistent with our previous conclusions: RAR showed a positive correlation with AKI as either a continuous (OR 1.46, 95% CI 1.22-1.76, P < 0.05) or categorical variable (OR 2.79, 95% CI 1.69-4.59, P < 0.05). The results are presented in Table 2 (Page 5, line 143-145). Regrettably, due to missing data, 24-hour fluid resuscitation volume and intra-abdominal pressure cannot be included in the study. We have noted this limitation in the revised manuscript (Page 8, line 222-225): Fourthly, factors with important clinical significance, such as fluid resuscitation volume within the first 24 hours and intra-abdominal pressure, could not be included in the study due to data missing in the database.

We hope these revisions meet your requirements.

Sincerely,

Chenyang Xu
