# Supplementary material for: Predictive value of red cell distribution width to albumin ratio for acute kidney injury in patients with acute pancreatitis
Source: PLoS One. 2026 Feb 27;21(2):e0341471. doi: 10.1371/journal.pone.0341471 (PMC12948089; doi:10.1371/journal.pone.0341471)
Supplement: Attachment — Submitted filename: Response_to_Reviewers_auresp_3.docx [file pone.0341471.s006.docx]

Dear Editors and Reviewers,

Thank you for handling our manuscript and providing constructive comments. We have carefully addressed the comments as follows:

**Additional Editor Comments:**

Q1: Here and there the grammar could use some correction. Please re-read the paper before publication. i.e.

a. The researches on the prognostic biomarkers of AP-AKI (67)
b. Restricted cubic spline (RCS) was performed (122)
c. or not-forprofit sectors. (249)

**Response:** We sincerely appreciate the comments on the grammar and expression of our manuscript. We have carefully re-read the entire paper, revised all identified grammatical errors and inappropriate expressions, and ensured the accuracy and consistency of the language.

Q2: Line 97: say something like: after applying filters, 600 patients were left.

**Response:** Thank you for your valuable suggestions. We have revised the the sentence as follows: After applying the exclusion criteria, 600 patients were finally included in the study (Line 97) .

**Reviewer #1 and Reviewer #2**

**Response:** Thank you for reviewing our manuscript and providing valuable comments.

**Reviewer #4**

Q1. The retrospective and observational nature of the study inherently limits causal inference. While the authors appropriately describe RAR as an “independent risk factor,” some language in the Discussion and Conclusion sections may still imply predictive or causal utility. I recommend further emphasizing that the findings demonstrate association rather than causation, particularly when discussing clinical implementation and “targeted interventions.”

**Response:** We thank the reviewer for this important reminder. We have carefully revised the Discussion and Conclusion sections to minimize and as far as possible eliminate language that could imply causal or definitive predictive utility of RAR.

Q2. The manuscript would benefit from clearer clarification regarding the exact timing of AKI occurrence relative to ICU admission. Whether RAR was measured strictly prior to AKI onset in all cases. This clarification is essential to support the interpretation of RAR as an early predictive biomarker rather than a marker of concurrent disease severity.

**Response:** We sincerely appreciate the reviewer’s critical insight regarding the need to clarify the timing of AKI occurrence relative to ICU admission and RAR measurement, which is essential to validate RAR as an early predictive biomarker for AKI in AP patients.

We acknowledge that this limitation stems from the retrospective nature of our study and inherent constraints of the clinical database utilized. Specifically, our data from the MIMIC database only includes the first measurement of RAR within 24 h of ICU admission and the first documentation of AKI following ICU admission, but it does not provide the exact hourly interval between RAR measurement and AKI onset for individual patients. Due to the de-identified and aggregated nature of the database, we are unable to retrieve additional granular data to confirm whether RAR was measured strictly prior to AKI onset in all cases.

To address this comment, we have supplemented the Limitations section of the manuscript with a detailed discussion (Line 224-228).

Q3. While ROC analysis shows that RAR has a higher AUC than RDW or albumin alone, the manuscript would benefit from a clearer discussion of whether the incremental improvement in AUC is clinically meaningful. This does not require additional analyses but should be addressed conceptually in the Discussion.

**Response:** We sincerely appreciate the reviewer’s insightful suggestion. As recommended, we have supplemented the Discussion section with a targeted conceptual elaboration on this issue as follow: Notably, ROC curve analysis demonstrated that RAR showed a higher AUC than either RDW or albumin alone for predicting AKI in patients with AP, and this superior AUC directly indicated its stronger discriminatory ability and clinical significance (Line 204).

Q4. The Methods section refers to “Cox regression analysis,” whereas the Results and tables primarily describe logistic regression. Please ensure consistency and clarify whether time-to-event analysis was actually performed.

**Response:** Thank you sincerely for your careful review and insightful comments on our manuscript. The term “Cox regression analysis” (Line 19) in the Methods section was an inadvertent error. We have revised it to logistic regression analysis to maintain consistency with the results and tables presented in the manuscript. No time-to-event analysis was performed in this study, and this has been clarified in the revised Methods section.

Q5. Figure legends could be more descriptive, particularly for the restricted cubic spline and ROC analyses.

**Response:** We thank the reviewer for this constructive suggestion. We have revised the legends for the figures involving restricted cubic spline (RCS) and receiver operating characteristic (ROC) analyses as follows:

**Figure 2** ROC curves for biomarkers to predict AKI. ROC curves of RAR (green line), Rdw (blue line), albumin (red line). RAR, red blood cell distribution width-to-albumin ratio; Rdw, red blood cell distribution.

**Figure 3** Restricted cubic spline curve of RAR with acute kidney injury incidence. RAR was entered as a continuous variable. Hazard ratios were adjusted for Gender, Age, Race, Wbc, Hct, CRRT, Map, Creatinine, Bun, Ast. RAR, red blood cell distribution width-to-albumin ratio; WBC, white blood cell; Hct, hematocrit; CRRT, continuous renal replacement therapy; MAP, mean arterial pressure; BUN, blood urea nitrogen; AST, aspartate aminotransferase. The red line represents the estimated values. The shaded area represents the corresponding 95% confidence intervals.

Q6. Minor Language and Clarity Issues:

a) Abstract line 28: "critical ill" should be "critically ill"

b) Line 74: "remains confirmed" should be "remains unconfirmed"

c) Line 231: "predictor for AKI for AKI" - remove duplicate

**Response:** We thank the reviewer for identifying these errors. All revisions have been made in the revised manuscript.

We hope these revisions meet your requirements.

Sincerely,

Chenyang Xu
